# Mutually Exclusive Expression of *COL11A1* by CAFs and Tumour Cells in a Large panCancer and a Salivary Gland Carcinoma Cohort

**DOI:** 10.1007/s12105-021-01370-0

**Published:** 2021-08-10

**Authors:** Christoph Arolt, Franziska Hoffmann, Lisa Nachtsheim, Philipp Wolber, Orlando Guntinas-Lichius, Reinhard Buettner, Ferdinand von Eggeling, Alexander Quaas, Jens Peter Klußmann

**Affiliations:** 1 Medical Faculty, Institute of Pathology, University of Cologne, Kerpener Straße 62, 50937 Cologne, Germany; 2 Department of Otorhinolaryngology, MALDI Imaging and Innovative Biophotonics, Jena University Hospital, 07747 Jena, Germany; 3 Department of Otorhinolaryngology, Head and Neck Surgery, Medical Faculty, University of Cologne, 50937 Cologne, Germany; 4 Department of Otorhinolaryngology, Head and Neck Surgery, Jena University Hospital, 07747 Jena, Germany; 5 MALDI Imaging, Core Unit Proteome Analysis, DFG Core Unit Jena Biophotonic and Imaging, Laboratory (JBIL), Jena University Hospital, 07747 Jena, Germany; 6 Medical Faculty, Centre for Molecular Medicine Cologne (CMMC), University of Cologne, 50937 Cologne, Germany

**Keywords:** *COL11A1*, CAFs, Salivary gland carcinoma, Tumour microenvironment, Extracellular matrix, RNA-ISH

## Abstract

Procollagen 11A1 (*COL11A1*) is a central component of the extracellular matrix in many carcinomas, which is considered to be mainly produced by cancer associated fibroblasts (CAFs). As *COL11A1* expression correlates with adverse prognosis and is implicated in chemoresistance, it is a promising putative target. For the first time, we used RNA in-situ hybridization to systematically identify the cells that produce *COL11A1* in the ten most prevalent carcinoma types, lymphomas (n = 275) and corresponding normal tissue (n = 55; panCancer cohort). Moreover, as most salivary gland carcinomas (SGC) display distinct stromal architectures, we also analysed 110 SGC. The corresponding protein formation of *COL11A1* was determined by MALDI-TOF–MS-Imaging. We report that colon, breast and salivary duct carcinomas are highly infiltrated by *COL11A1* positive CAFs (CAFs_*COL11A1*_) and might thus be promising candidates for antidesmoplastic or *COL11A1*-targeted therapies. The amount of CAFs_*COL11A1*_ correlated significantly with tumour grade, tumour stage and nodal spread in the panCancer cohort. Significant associations between CAFs_*COL11A1*_ and vascular invasion, perineural spread and nodal spread were observed in the SGC cohort. Also, we discovered that tumour cells of intercalated duct derived SGC and CAFs produce *COL11A1* in a mutually exclusive manner. Our findings represent a novel mode of extracellular matrix production in carcinomas and could be highly relevant in the future. Our findings elucidate the mode of *COL11A1* expression in very different carcinoma types and may aid to categorise tumours in the setting of possible future *COL11A1*-related therapies.

**Supplementary Information:**

The online version contains supplementary material available at 10.1007/s12105-021-01370-0.

## Introduction

As the efficacy of mutation-specific therapies is limited by resistance mechanisms, more recently, the interest of cancer research has expanded towards the non-neoplastic tumour microenvironment (TME). It provides tumour promoting growth factors, an aberrant neo-vasculature and an immune milieu that supports the growth of neoplastic cells [1–4]. More than 50% of the tumour surface can be attributed to the desmoplastic stroma, a highly specialised extracellular matrix (ECM) [5] which is considered to be secreted by cancer associated fibroblasts (CAFs) [6, 7]. Several agents that target either CAFs or the ECM are currently being trialed [8].

Procollagen 11A1 (*COL11A1*), the α1 chain of collagen XI, has been found to be consistently upregulated in the ECM of different carcinoma types [9]. Also, an overexpression of *COL11A1* has been associated with an adverse outcome in a variety of primaries including breast (MC) [10], colorectum (CRC) [9], ovary (OC) [9, 11], lung [12], bladder [13], kidney [14] and pancreas (PDAC) [15]. García-Pravia et al. and Jia et al. revealed that in PDAC and CRC, this overexpression of *COL11A1* can be traced back to intratumoral CAFs [9, 16]. Physiologically, *COL11A1* is expressed in cartilaginous tissues and mesenchymal stem cells while it is nearly undetectable in other normal tissues including resident fibroblasts and most benign sclerotic conditions [9, 17–20]. This relative specificity is an advantage over other fibroblast markers such as aSMA, PDFGRß or FAP and could make *COL11A1* a more reliable indicator for a CAF phenotype [20, 21]. Also, the expression of *COL11A1* has been associated with upregulation pathways that are typically active in CAFs [20]. Also more recently, Wu et al. and Nallantighal et al. [22, 23] have mechanistically demonstrated that *COL11A1* expression can lead to chemoresistance by the induction of apoptosis inhibitor proteins [11] and fatty acid oxidase [22]. Thus, *COL11A1* is a promising candidate for targeted therapy.

Salivary gland carcinomas (SGC) are a group of rare and heterogenous tumour entities. Most salivary gland carcinoma subtypes exhibit pathognomonic growth patterns and sometimes entity-defining gene translocations [24]. This group of carcinomas makes up the vast majority of SGC as it comprises the most prevalent subtypes such as adenoid cystic carcinomas (AdCy) and mucoepidermoid carcinomas (MuEp). Unfortunately, most entity-specific gene translocations cannot yet be therapeutically targeted. The lack of options for efficient systemic therapy has prompted the exploration of the TME of SGC. While the presence of several immune checkpoint molecules has been recently reported [25–27], the extracellular matrix or the presence of CAFs in the TME of SGC has not yet been systematically addressed.

Recently, we have discovered that in SGC, *COL11A1* is not only expressed by CAFs but also by AdCy tumour cells [28]. As *COL11A1* is a promising therapeutic target due to its involvement in chemoresistance, in the present study, we used RNA in-situ staining to determine the frequency of *COL11A1* positive CAFs (CAFs_*COL11A1*_) and tumour cells in a large cohort of different SGC and the ten most prevalent carcinoma primaries on tissue microarrays (TMA). We reveal that SGC arising from the excretory duct display higher frequencies of CAFs_*COL11A1*_ than any other primary we analysed. Of note, *COL11A1* expression by tumour cells was nearly exclusive to SGC that are derived from the intercalated duct or the acini. Our results indicate that therapeutic targeting of *COL11A1* might have a particularly high potential in SGC.

## Materials and Methods

### Recruitment of Patients and TMA Preparation

For the SGC cohort, all formalin-fixed paraffin-embedded (FFPE) SGC specimen, which were resected at the Department of Oto-Rhino-Laryngology, Head and Neck Surgery of the University of Cologne between 1998 and 2018 were retrieved from the archives of the Institute of Pathology, University Hospital of Cologne (n = 110). All cases were revised in detail by two pathologists (CA, AQ). Immunohistochemical staining for CK7, p63, NOR-1, S100, androgen receptor and HER2 as well as FISH break-apart analysis for *MYB*, *MYBL1*, *MAML2* and *ETV6* genes were employed in case the original diagnosis would not be unequivocal. For the panCancer cohort, 25 cases of each tumour type as well as five tissue blocks with the respective normal tissue were retrieved from the archives. These cases comprised the ten most prevalent carcinoma types according to the US National Cancer Institute [29] as well as the different lymphomas (Table S1). Cases were selected in order to mirror the actual relative prevalence of each grade and tumour subtype (e.g., growth pattern, ER positivity in case of breast carcinomas) while also including rare forms. If available, basic clinicopathological data as sex, age and TNM classification were recorded. Before TMA preparation, it was ensured that sufficient residual tumour material for possible complementary diagnostic tests would be available afterwards. TMAs were prepared as described before [25]. Briefly, four tissue cylinders in case of the SGC cohort and two tissue cylinders in case of the panCancer cohort were punched out from FFPE block and transferred to an empty paraffin block. All procedures were in accordance with the ethical standards of the University of Cologne and the Helsinki Declaration from 1975 and its revision in 1983. Patients gave their written informed consent to participate in this study.

### RNA In-Situ Hybridization (RNA-ISH) and Immunohistochemistry (IHC)

The RNAscope assays were performed according to the manufacturer’s instruction, as previously reported [30]. Briefly, 5 μm sections were cut from FFPE blocks, pre-treated (30 min for pre-treatment 2 and 3), digested and hybridised at 40 °C with mRNA probes specific for human *COL11A1* (each ready to use, Advanced Cell Diagnostics, Bio-Techne, Minneapolis, MN, USA) using a Ventana Discovery system (Roche, Switzerland). Subsequently, the slides were counterstained for 10 s in haematoxylin. Immunohistochemical staining of CD8 + T cells and p53 mutation status was carried out before for this cohort. Tumours were termed “inflamed” if the amount of CD8 + T cells exceeded > 100 per core [25].

### Semiquantitative Scoring of CAFs_***COL11A1***_

CAFs_*COL11A1*_ in the tumour tissue were morphologically identified. The percentage of *COL11A1*-positive stromal surface as indicator for CAFs_*COL11A1*_ was recorded by two pathologists (C.A. and A.Q.) as follows: 0: < 1%; 1: 1–20%; 2: 21–50%; 3: > 50%; 4: > 50% and high staining intensity due to frequently merged RNA-ISH signals. As two (panCancer cohort) and four (SGC cohort) TMA-cores of 1 mm diameter were scored per case, the upper median was chosen as resulting score for further statistical analysis. The percentage of TC_*COL11A1*_ was noted for each spot and the mean percentage was calculated. For binary analyses, cases with CAFs_*COL11A1*_ score ≥ 1 or ≥ 1% TC_*COL11A1*_ were designated stroma-positive or tumour cell-positive, respectively.

### Detection of COL11A1 Protein by MALDI-TOF–MS-Imaging (MSI)

For the detection of the protein expression, TMAs with samples from 41 patients of the SGC cohort and from 100 patients of the panCancer cohort (breast, prostate, lung, colorectum n = 25 each) were used. Sample preparation, MSI data acquisition as well as data and imaging analysis were performed as previously published [28]. We detected one *COL11A1* peptide after tryptic cleavage [(R)GEKGEAGPPGAAGPPGAK(G); m/z value: 1545.82]. From this peptide, four adducts, [M + H] + (1546.828 m/z), [M + NH4] + (1563.854 m/z), [M + Na] + (1568.81 m/z) and [M + K] + (1584.784 m/z) were recognised. As all adducts were distributed equally among the different tumour types, only the measurements for the [M + H] + adduct were used for further comparisons.

### Statistical Analysis

Statistical analysis was carried out with SPSS statistical software (IBM SPSS 25.0, Armonk, NY). Bar plots and dot plots were drawn with RStudio (R Foundation for Statistical Computing, Vienna, Austria.) and the R package ggplot2 [31]. Interdependencies between categorical variables were tested using Fisher’s exact test or Pearson’s Chi-square test depending of the group size. Correlations between ordinal variables were calculated with spearman’s rank correlation coefficient. A Kruskal–Wallis-Test with p value correction was employed for the comparisons of COL11A1 protein expression between different tumour types. P values below 0.05 were considered significant.

## Results

### The Highest Frequency of CAFs_***COL11A1***_ can be observed in SGC, MC and Colon CRC

**Fig. 1:**
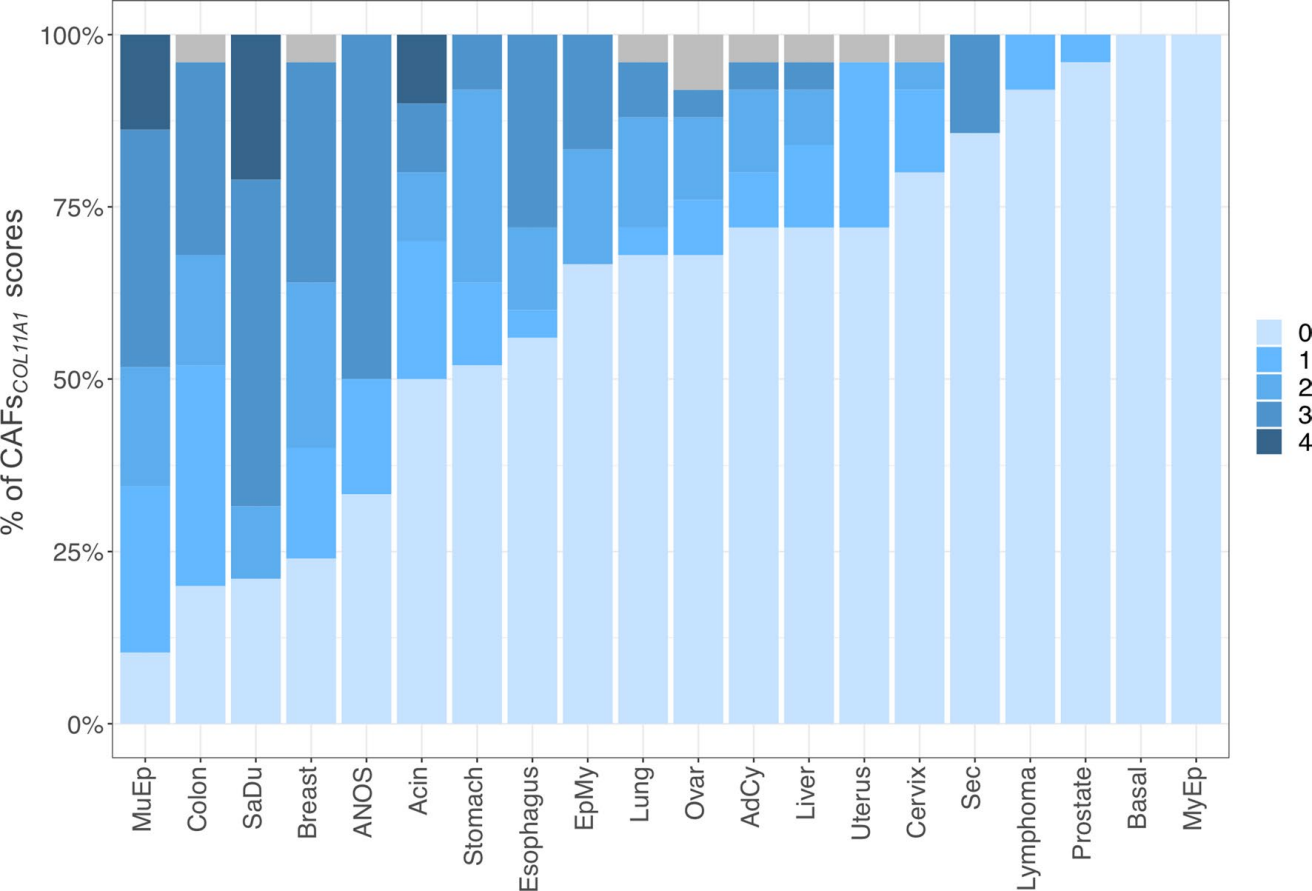
Relative distribution of stromal *CAFs*_*COL11A1*_ scores among different tumour types. Stacked bar chart, visualizing the relative distribution of *CAFs*_*COL11A1*_ scores in the analysed tumour types. Both the panCancer and the SGC collective are depicted. *CAFs*_*COL11A1*_ scores are color-coded as indicated on the right-hand side. Cases that were not analysable are marked in grey. *MuEp* mucoepidermoid, *SaDu* salivary duct, *ANOS* adenocarcinoma NOS, *EpMy* epithelial-myoepithelial, *AdCy* adenoid cystic, *Sec* secretory, *Basal* basal cell, *MyEp* myoepithelial

The panCancer cohort (n = 275) comprised the ten most prevalent carcinoma types as well as lymphomas (25 cases each). Additionally, five samples of normal tissue of each respective organ were included (n = 55). We measured the infiltration by CAFs_*COL11A1*_ using a semiquantitative score (Score 0—4) that reflected the percentage of stromal surface stained by the *COL11A1* RNA-ISH. Additionally, we assessed the percentage of tumour cells with *COL11A1* staining (TC_*COL11A1*_). CAFs_*COL11A1*_ were almost exclusively observed in tumour tissues with an overall positivity rate of 34.5% compared to 1.6% in normal tissue (one positive endometrium sample). We observed dramatic differences of CAFs_*COL11A1*_ infiltration across different primary sites (Fig. 1 and Table S1). The highest frequencies of positive tumours were noted for CRC (79%), MC (75%), gastric (48%) and esophageal carcinomas (44%), while only 8% and 4% of lymphomas and prostate carcinomas were infiltrated by CAFs_*COL11A1*_. Interestingly, CRC, MC and esophageal carcinomas exhibited very similar frequencies of highly positive cases (Score 3), reaching 29.2%, 33.3% and 28%, respectively. Conversely, only 8% of all stomach carcinomas were assigned to this category. None of the cases from the panCancer cohort exhibited confluent stromal *COL11A1* staining required for the maximum CAFs_*COL11A1*_ Score of 4, which was observed in several SGC subgroups.

When all cases of the panCancer cohort were pooled (Table 1), the CAFs_*COL11A1*_ score strongly correlated with tumour grade (p = 0.001) and tumour stage (p < 0.001). To a lesser extent, but still significantly, CAFs_*COL11A1*_ score also correlated with nodal spread (p = 0.048). Moreover, a trend for a higher rate of lymphatic invasion and higher age and was observed for tumours with infiltration by CAFs_*COL11A1*_ (p values: 0.067 and 0.081, respectively). The trend for a higher percentage of CAFs_*COL11A1*_ infiltrated carcinomas among females (p = 0.078) resulted from the high percentage of MCs.

**Table 1:** Percentage of cases with *CAFs*_*COL11A1*_ and clinicopathological features of the panCancer cohort

	*CAFs*_*COL11A1*_ score				p	n	Spearman coefficient
	0	1	2	3			
Grade							
G1	85.0%	10.0%	2.5%	2.5%	**0.001**	242	0.226
G2	65.7%	9.8%	8.8%	15.7%			
G3	51.0%	18.0%	20.0%	11.0%			
T							
pT1	78.0%	10.0%	6.0%	6.0%	** < 0.001**	242	0.264
pT2	58.1%	8.1%	16.1%	17.7%			
pT3	47.5%	23.0%	16.4%	13.1%			
pT4	47.4%	15.8%	21.1%	15.8%			
N							
pN0	61.3%	10.4%	11.3%	17.0%	**0.048**	172	0.152
pN1	58.5%	17.1%	12.2%	12.2%			
pN2	17.6%	29.4%	29.4%	23.5%			
pN3	37.5%	0.0%	62.5%	0.0%			
Total	55.2 (95)	13.4 (23)	15.7 (27)	15.7 (27)			

	Negative		Positive		p	n	
Lineage							
Adeno	61.1%		38.9%		0.634*	242	
Squamous	63.3%		36.7%				
Neuroend	66.7%		33.3%				
Liver cell	75.0%		25.0%				
pT							
< pT3	70.4%		29.6%		**0.001#**	242	
≥ pT3	47.5%		52.5%				
pN							
pN −	61.3%		38.7%		*0.06#*	173	
pN +	46.3%		53.7%				
V							
V0	61.6%		38.4%		0.245#	222	
V1	50.0%		50.0%				
L							
L0	65.7%		34.3%		*0.067#*	222	
L1	52.9%		47.1%				
Pn							
Pn0	64.4%		35.6%		0.828#	95	
Pn1	61.1%		38.9%				
Age							
< 65 years	68.8%		31.3%		*0.081#*	238	
≥ 65 years	57.1%		42.9%				
Sex							
Women	58.2%		41.8%		0.078*	240	
Men	69.7%		30.3%				
Total	62.8 (152)		37.2 (90)				

Upper half: *CAFs*_*COL11A1*_ score in relation to ordinally scaled pathological attributes. Lower half: Comparison of negative (*CAFs*_*COL11A1*_ score = 0) and positive (*CAFs*_*COL11A1*_ score > 0) cases with respect to nominally scaled clinicopathological parameters. Given as percentage, absolute numbers in brackets. P values below 0.05 are indicated in bold type, p values below 0.1 are marked in italic

*V* vascular invasion, *L* lymphatic invasion, *Pn* perineural spread

#Chi-square test

*Fisher’s exact test (lower half)

MC and CRC were infiltrated by CAFs_*COL11A1*_ in over 50% of the cases and were consecutively studied in more detail. For MC (Table S2), a significant positive correlation with stromal CAFs_*COL11A1*_ score was observed for tumour grade and Ki67 index (0.478, p = 0.018 and 0.656, p = 0.002, respectively). Also, the presence of CAFs_*COL11A1*_ was associated with negativity for oestrogen receptor (ER), as 100% of ER- and only 54.5% of ER + MC were infiltrated by CAFs_*COL11A1*_ (p = 0.011). In this subgroup analysis, no significant dependency between CAFs_*COL11A1*_ score and progesterone or androgen receptor status or clinicopathological criteria was observed. Also, no significant association between CAFs_*COL11A1*_ score and clinicopathological parameters, including microsatellite instability was observed for CRC. Of note, tumour cells with staining for *COL11A1* (TC_*COL11A1*_*)* were detected in one OC as well as one endometrial carcinoma with a percentage of 5% and 70% of tumour cells, respectively.

### CAF Based *COL11A1* Expression Varies Considerably Among SGC Types

**Fig. 2:**
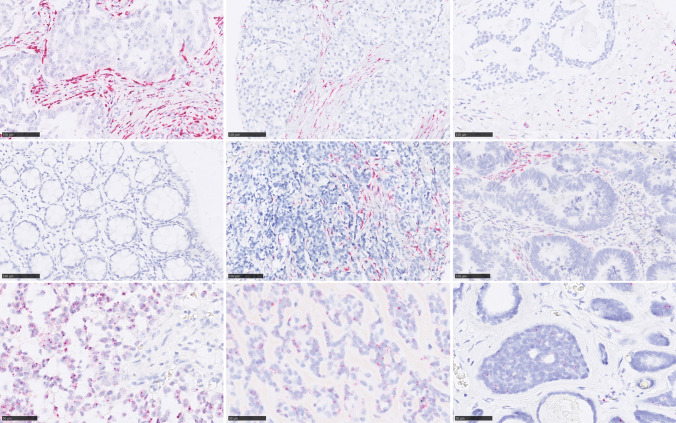
COL11A1 mRNA expression by CAFs (CAFs_COL11A1_) and tumour cells (TC_COL11A1_). Top Row: CAFs_COL11A1_ in SaDu (Score 4), MuEp (Score 3) and AdCy (Score 2). Middle row: CAFs_COL11A1_ in normal colon mucosa (Score 0), ER + breast carcinoma (Score 3) and colon carcinoma (Score 2). Bottom row: TC_COL11A1_ in MyEp (90%), EpMy (80%), AdCy (50%). 400x, reference bar: 50 µm

110 SGC were available for *COL11A1* mRNA-ISH, containing 29 MuEp, 25 AdCy, 19 salivary duct carcinomas (SaDu), 10 acinic cell carcinomas (Acin), 7 secretory carcinomas (Sec), 6 epithelial-myoepithelial carcinomas (EpMy), 6 adenocarcinomas not otherwise specified (ANOS) as well as 4 basal cell (Bas) and 4 myoepithelial carcinomas (MyEp). We observed CAFs_*COL11A1*_ in the peritumoural stroma with marked differences across the different subtypes (Figs. 1 and 2). While frequencies of more or equal to 50% of cases with CAFs_*COL11A1*_ were detected among MuEp (89.7%), SaDu (78.9%) and Acin (50.0%), MyEp and Bas showed no CAFs_*COL11A1*_ at all. A heterogeneous distribution was observed in EpMy (33.3%), AdCy (28.0%) and Sec (14.3%). A CAFs_*COL11A1*_ score of 4 was only detected among MuEp and SaDu—none of the tumours from other organs exhibited such intense staining.

When all SGC cases were pooled, CAFs_*COL11A1*_ score positively correlated with higher N-status and CD8 + T cell infiltration (p = 0.015 and 0.017, respectively, Table 2). Interestingly, all cases with vascular invasion by the tumour cells also exhibited CAFs_*COL11A1*_ (p = 0.004). Moreover, the presence of CAFs_*COL11A1*_ was associated with perineural spread (p = 0.004). No association between CAFs_*COL11A1*_ and either tumour grade or pT-status was observed in the SGC cohort. On single-entity level, AdCy with CAFs_*COL11A1*_ were more likely to exhibit perineural spread (p = 0.007) or a TP53 mutation (p = 0.003; Table S3). No other significant associations of CAFs_*COL11A1*_ with clinicopathological parameters were observed in the other tumour types.

**Table 2:** Percentage of cases with CAFs_COL11A1_ and clinicopathological features of the SGC cohort

	*CAFs*_*COL11A1*_ score					p	n	Spearman coefficient
	0	1	2	3	4			
Grade								
G1	32.0%	16.0%	12.0%	24.0%	16.0%	0.754	72	− 0.37
G2	40.9%	0.0%	13.6%	36.4%	9.1%			
G3	32.0%	16.0%	16.0%	28.0%	8.0%			
pT								
pT1	54.2%	12.5%	12.5%	16.7%	4.2%	0.209	104	0.125
pT2	40.7%	22.2%	7.4%	25.9%	3.7%			
pT3	52.2%	0.0%	4.3%	21.7%	21.7%			
pT4a	47.6%	4.8%	19.0%	23.8%	4.8%			
pT4b	22.2%	22.2%	11.1%	33.3%	11.1%			
N								
pN0	52.3%	12.3%	9.2%	20.0%	6.2%	**0.015**	102	0.239
pN1	44.4%	22.2%	11.1%	22.2%	0.0%			
pN2	28.6%	7.1%	14.3%	32.1%	17.9%			
CD8								
0	55.6%	11.1%	14.8%	18.5%	0.0%	**0.017**	109	0.228
1	48.1%	13.0%	7.4%	24.1%	7.4%			
2	32.1%	7.1%	14.3%	28.6%	17.9%			
Total	45.9 (50)	11.0 (12)	11.0 (12)	23.9 (26)	8.3 (9)			

	Negative		Positive			p	n	
pT								
< pT3	46.2%		53.8%			1#	105	
≥ pT3	45.3%		54.7%					
pN								
pN −	51.5%		48.5%			*0.067#*	103	
pN +	32.4%		67.6%					
V								
V0	48.8%		51.2%			0.004*	91	
V1	0.0%		100.0%					
L								
L0	46.9%		53.1%			0.334*	92	
L1	27.3%		72.7%					
Pn								
Pn0	56.1%		43.9%			**0.004#**	96	
Pn1	23.3%		76.7%					
Age								
< 65y	51.4%		48.6%			0.102#	110	
≥ 65y	33.3%		66.7%					
Sex								
Women	45.9%		54.1%			1#	110	
Men	44.9%		55.1%					
TP53								
Wildtype	51.4%		48.6%			*0.088#*	106	
Mutated	31.3%		68.8%					
CD8								
Not inflamed	50.0%		50.0%			0.126#	110	
Inflamed	32.1%		67.9%					
Total	45.5 (50)		54.5 (60)					

Upper half: CAFs_*COL11A1*_ score in relation to ordinally scaled pathological attributes. Comparison of negative (CAFs_*COL11A1*_ score = 0) and positive (CAFs_*COL11A1*_ score > 0) cases with respect to nominally scaled clinicopathological parameters, given as percentage, absolute numbers in brackets. P values below 0.05 are indicated in bold type, p values below 0.1 are marked in italic

*V* vascular invasion, *L* lymphatic invasion, *Pn* perineural spread

#Chi-square test

*Fisher’s exact test (lower half)

For 14 cases, both the primary and a nodal metastasis were analysed (Table S4). In 50% of the cases, both the primary and the metastasis were either positive or negative for CAFs_*COL11A1*_. 35.7% were positive in the primary but not in the nodal metastasis while in the opposite was the case in 14.3%.

### Tumour Cell Based *COL11A1* Expression is Restricted to SGC with Intercalated Duct Origin and Varies Markedly Among the Different Histotypes

**Fig. 3:**
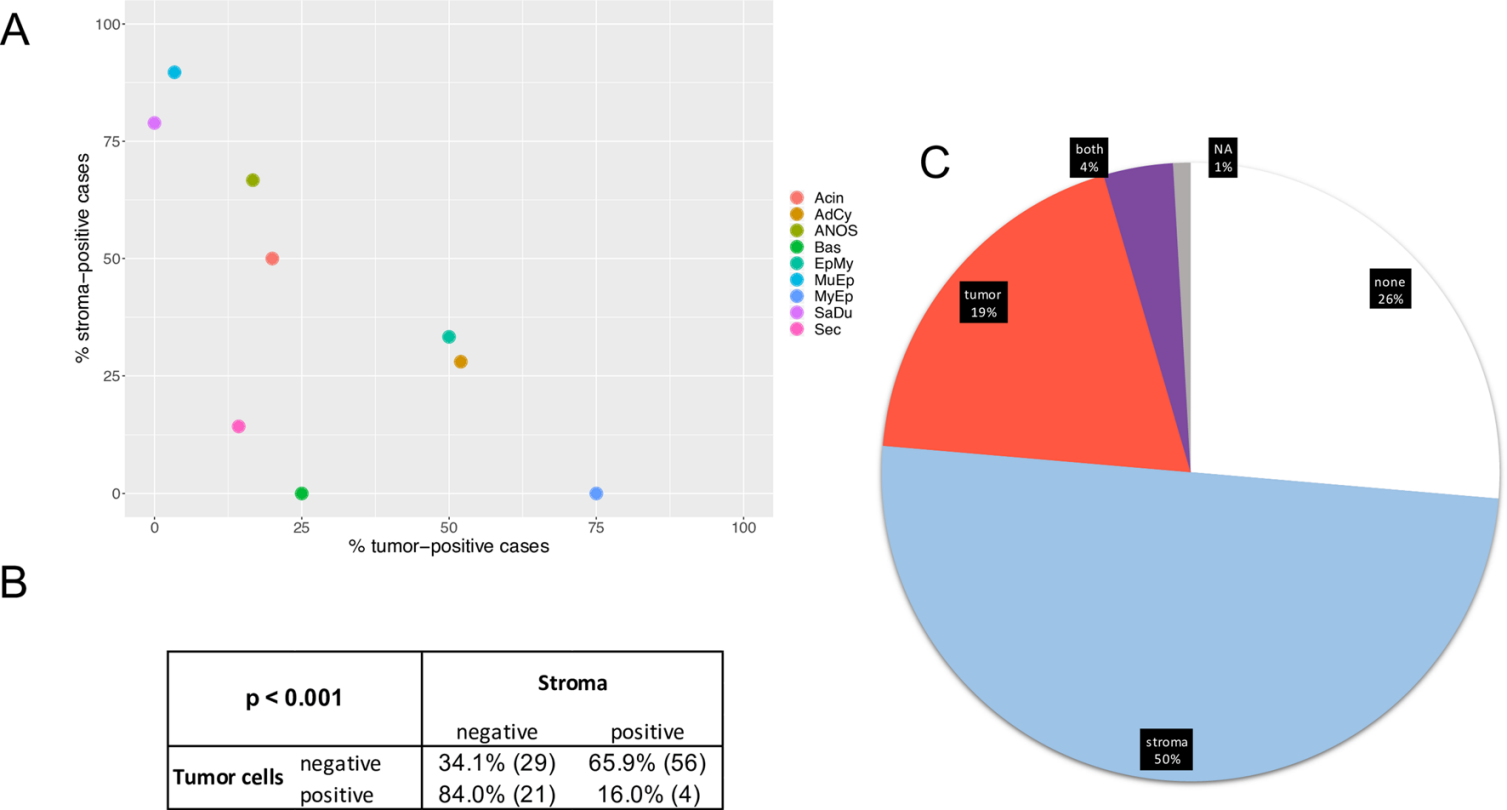
Comparison of SGC positive for CAFs_COL11A1_ and TC_COL11A1_ in SGC. **A** Dotplot, locating each carcinoma entity in a bidimensional manner according to the respective percentage of cases with TC_COL11A1_ (x axis) and CAFs_COL11A1_ (y axis). **B** Table demonstrating an anti-proportional pattern of COL11A1 expression in the above-mentioned tumour compartments. **C** Pie chart, illustrating the percentage of cases with CAFs_COL11A1_, TC_COL11A1_ or combined staining

In addition to a staining of CAFs, some SGC types also exhibited TC_*COL11A1*_. The highest rate of tumours with TC_*COL11A1*_ was observed among MyEp (75.0%, n = 4), AdCy (52.0%, n = 25) and EpMy (50.0%, n = 6). Similarly, the mean percentage of TC_*COL11A1*_ in individual tumours was highest in MyEp followed by EpMy and AdCy (Table S5). Conversely, these entities showed low frequencies of cases with CAFs_*COL11A1*_ of 0.0%, 28.0% and 33.3%, respectively (Fig. 3A). As expected, TC_*COL11A1*_were inversely correlated with the presence of CAFs_*COL11A1*_ (p < 0.001, Fig. 3B). This nearly mutually exclusive pattern was sustained by the fact, that only 4% of all cases were positive for both tumour cell and stromal *COL11A1*, while 69% stained exclusively for either of the two patterns (Fig. 3C).

Neither stromal nor intratumoural COL11A1 staining was associated with an alteration of 5-year event-free survival.

### While a Parallel Expression of COL11A1 Protein and RNA is Observed in most Tumour Types, in AdCy, Protein Abundance Markedly Exceeds RNA Expression

**Fig. 4:**
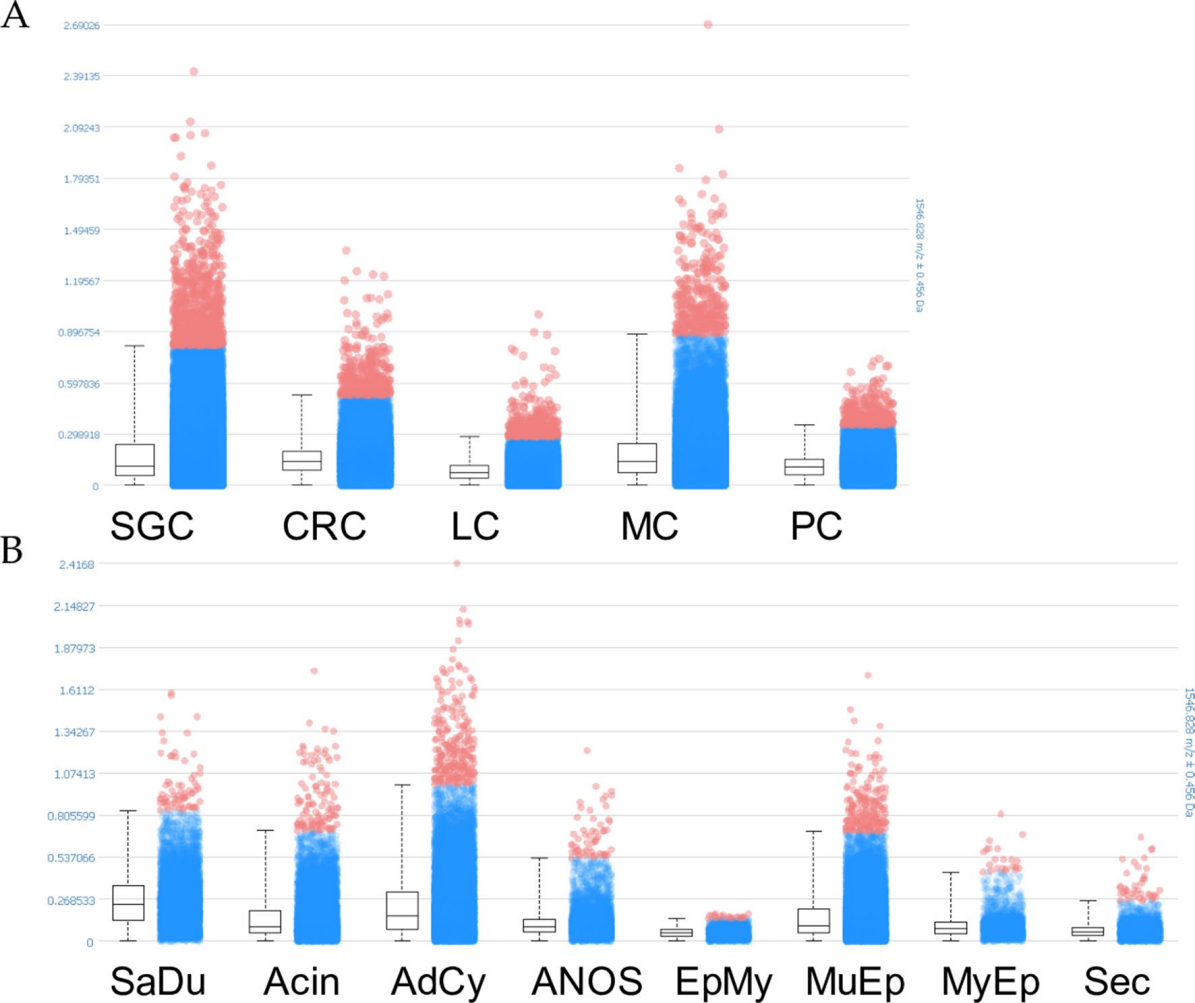
Distribution of one COL11A1 peptide in TMA sections. Intensity box plots comparing the intensities of the shown m/z value for different primary sites (**A**) and SGC types (**B**). The horizontal line of the box part represents the mean intensity of the m/z value measured over all pixels. The blue dots represent pixel with spectra in which intensities of the m/z value are between the lower and upper quantile. The red dots represent pixel with spectra outside of these intervals. *SGC* salivary gland carcinoma, *CRC* colorectal carcinoma, *LC* lung carcinoma, *MC* mamma carcinoma, *PC* prostate carcinoma, *SaDu* salivary duct carcinoma, *Acin* acinic cell carcinoma, *ANOS* adenocarcinoma not-otherwise-specific, *EpMy* epithelial-myoepithelial carcinoma, *MuEp* mucoepidermoid carcinoma, *MyEp* myoepithelial carcinoma, *Sec* secretory carcinoma

Using MSI, COL11A1 protein was measured in TMA tissue samples from both cohorts. After tryptic cleavage, we detected one COL11A1 peptide [(R)GEKGEAGPPGAAGPPGAK(G); m/z value: 1545.82]. Among the different primary sites, the highest intensities were noted for SGC, breast and colon carcinomas, which largely confirmed our results from the RNA-ISH analysis (Fig. 4A). When comparing the different SGC types, the highest intensities for COL11A1 protein were measured in SaDu and AdCy, resulting in a significant difference to the other histologic groups (p value < 0.001) (Fig. 4B). Even though this finding supports the notion that COL11A1 is markedly upregulated in SaDu, it is remarkable that the moderate frequency of CAFs_*COL11A1*_ and TC_*COL11A1*_ in AdCy results in such pronounced protein expression. This seeming discrepancy between ISH and MSI results might be traced back to a slow turnover of COL11A1 in AdCy, possibly due to a relative absence of CAFs in these tumours.

## Discussion

Recently, we profiled the expression of several ECM genes in SGC and discovered that *COL11A1* is part of an ECM gene signature which is distinctively upregulated in SaDu [28]. Here, for the first time we assess the frequency of CAFs_*COL11A1*_ in a large group of 110 salivary gland carcinomas and another cohort which comprises the ten most frequent carcinoma types, lymphomas, and corresponding normal tissue. While an overexpression of *COL11A1* has been described for several primaries using bulk expression data, a systematic evaluation of CAFs_*COL11A1*_ with RNA-ISH has not yet been performed in any tumour type.

Within the panCancer cohort, the highest frequencies of CAFs_*COL11A1*_ and the highest amount of COL11A1 protein was detected in CRC and MC, while *COL11A1* RNA expression in normal tissue was largely absent. Several studies have revealed that a stromal expression of *COL11A1* is implicated in the malignant transition in these cancer types [10, 17, 32]. We show for the first time that this overexpression of *COL11A1* in MC can be exclusively attributed to *COL11A1* production by CAFs_*COL11A1*_ in the peritumoural stroma. Concordantly, we obtained very similar results for all other carcinomas from various sites in the panCancer cohort. In fact, only one endometrial and one OC displayed tumour cell based *COL11A1* production.

We found that the presence of CAFs_*COL11A1*_ in the peritumoural stroma correlated with higher tumour grade, T and N classification in the panCancer cohort. Although follow up data was not available, these results are in line with previous studies reporting a correlation between bulk *COL11A1* expression and an adverse outcome for many carcinomas [9, 11, 15, 33]. In accordance with Halsted et al. [32], we found that stromal *COL11A1* expression correlated with higher grade and ER negativity in MC. These, as well as a high ki67 index which also correlated with CAFs_*COL11A1*_ are all recognised predictors of poor outcome [34, 35]. In summary, our findings are in line with Toss et al. [10] who revealed that COL11A1 protein expression is associated with adverse prognosis in MC.

While the expression of *COL11A1* by CAFs has widely been accepted, it remains controversial whether carcinoma cells also produce *COL11A1*. Some authors have reported COL11A1 protein expression by tumours cells by analysing carcinoma cell lines [12, 20, 23] or cancer tissues with IHC [10, 23]. Surprisingly, only few studies have interrogated the mRNA expression in situ by RNA-ISH. Cheon et al. have used both IHC and RNA-ISH on serial sections in OC and found that *COL11A1* was nearly exclusively expressed by CAFs. Having compared both methods directly, they reported a higher cellular resolution for RNA-ISH [36]. Other authors have reported similar results for OC [9] and gastric carcinoma [37]. In line with these findings, we only detected TC_*COL11A1*_ in one OC and one endometrial carcinoma. This data indicates that tumour cell based *COL11A1* expression among the most prevalent carcinoma types is at least minor if not negligible. In our opinion, RNA-ISH assays rather than IHC should be used to validate the cellular origin of ECM proteins in tumour tissue and cell lines.

The so-called desmoplastic stroma is characterised by an increased stiffness due to deposition and crosslinking of collagens [38, 39]. These altered mechanic properties result in protumourigenic signalling through integrin-mediated mechanotransduction and a reduction of tissue perfusion with decreased bioavailability of antineoplastic agents [40, 41]. Jia et al. discovered that COL11A1 is a central component of a stromal pan-cancer gene signature. FAP, another CAFs marker, fibronectin and four collagens were among the ten genes most highly correlated with *COL11A1* [9]. Thus, the expression of *COL11A1* might additionally predict the presence of other collagens, which are major contributors to the ECM. Since the desmoplastic stroma is investigated as therapeutic target, diagnostic tests to quantify the amount of ECM deposition might be warranted in the future. Regarding the strong correlation of *COL11A1* expression with other ECM components, COL11A1-ISH might be a predictor for future anti-desmoplastic therapies.

Moreover, several studies have recently revealed that *COL11A1* mediated processes might be a promising therapeutic target by themselves. *COL11A1* is upregulated in chemoresistant carcinomas [12, 42] including OC [36] and has subsequently been suspected to be functionally involved in the process. Rada et al. mechanistically demonstrated that COL11A1 inhibits tumour cell apoptosis by activation of the Src-PI3K/Akt-NF-kB pathway [11]. Nallantighal et al. found out that *COL11A1* upregulates fatty acid oxidase (FAO) which confers cisplatin resistance [22]. The fact that FAO inhibitors, which have been approved for the therapy of cardiac disease [43, 44], also display an antineoplastic effect in-vitro and in-vivo makes them interesting candidates for anticancer therapy [45–47]. Thus, COL11A1 might be a feasible biomarker for a FAO-inhibitor therapy.

The frequency of CAFs_*COL11A1*_ varied dramatically among the different SGC subtypes. SaDu displayed the highest frequencies of scores 3 and 4, the latter being a staining intensity that has not been observed in any of the tumours from the panCancer cohort. Since SaDu have many overlapping features with ductal MC, it is not surprising that SaDu and MC are both characterised by high frequencies of CAFs_*COL11A1*_ and an absence of TC_*COL11A1*_. Together, our results provide a strong rationale to further investigate the potential of anti-desmoplastic therapies in SaDu and MC.

Interestingly, we found that the rate of CAFs_*COL11A1*_ positive AdCy cases was significantly higher among TP53 mutated tumours. The fact that p53 mutation is an adverse prognosticator for AdCy [48] further supports our notion that CAFs_*COL11A1*_ are associated to surrogates of poor outcome. As some CAF subtypes have been attributed immunomodulatory properties, we also scored the number of CD8 + T-lymphocytes but did not find a correlation with CAFs_*COL11A1*_. This is in line with current evidence, that immunomodulatory functions are exerted by specialised iCAFs rather than by mCAFs which are instead involved in ECM production [49, 50].

Ohtomo et al. found that SGC which derive from the acini (Acin, Sec) and the intercalated duct (AdCy, MyEp, Bas, EpMy) express the neural crest transcription factor SOX10, while tumours emerging from the excretory duct (SaDu, MuEp) do not [51]. Strikingly, we discovered that while *COL11A1* expression in the latter is nearly restricted to CAFs_*COL11A1*_ in their vicinity, SOX10 + tumours produce *COL11A1* in the tumour cells at varying rates. Interestingly, the mode of *COL11A1* production was nearly mutually exclusive as only 4% of the tumours exhibited both CAFs_*COL11A1*_ and TC_*COL11A1*_. This suggests that tumours with TC_*COL11A1*_ “do not require” CAFs_*COL11A1*_ in their TME. Together with the fact that 73% of all SGC produced *COL11A1* in either way, it indicates that *COL11A1* might play a major role in SGC tumourigenesis. Interestingly, we revealed that while the RNA expression of *COL11A1* in AdCy was not particularly high, the protein deposition in the tumours was marked. We speculate, that this seeming discrepancy might be traced back to a slow turnover of COL11A1 in AdCy, possibly due to a relative absence of CAFs in these tumours. As outlined above, *COL11A1* production strongly correlates with expression of several other ECM molecules. Thus, we hypothesise that SOX10 + SGC cells might produce most of their ECM themselves. Even though *COL11A1* production by tumour cells has been reported for mesenchymal neoplasms [20], we are the first to report this mechanism in carcinomas. These fundamental differences in the mode of *COL11A1* production might have implications on the effectiveness of COL11A1- and FAO-targeting drugs and might thus impact clinical decision making in the future.

The descriptive character of this study does not allow for mechanistic conclusions concerning the function of COL11A1. Moreover, as the size of each individual tumour group was rather small, correlations with clinicopathological parameters must be interpreted with caution and should be further validated in larger, individual studies.

We systematically assessed the frequency of CAFs_*COL11A1*_ and TC_*COL11A1*_ in the most prevalent carcinoma types and SGC. We report that (1) MC, CRC and SaDu do not produce *COL11A1* themselves but are highly infiltrated by CAFs_*COL11A1*_ and might thus be promising candidates for antidesmoplastic or *COL11A1*-targeted therapies. (2) *COL11A1* is produced by CAFs_*COL11A1*_ and intercalated duct SGC cells in a mutually exclusive manner which represents a novel mode of ECM production in carcinomas. (3) Finally, we propose a 4-tiered RNA-ISH-based scoring system for CAFs_*COL11A1*_ which could be highly relevant for future ECM or *COL11A1*-targeted therapies.

## Supplementary Information

Below is the link to the electronic supplementary material.

Supplementary file1 (PDF 263 kb)

## Data Availability

All primary data of the semiquantitative RNA-ISH analysis are available on request.

## Abbreviations

*COL11A1*: Procollagen 11A1
CAFs: Cancer associated fibroblasts
SGC: Salivary gland carcinomas
CAFs_*COL11A1*_: *COL11A1* positive CAFs
TME: Tumour microenvironment
ECM: Extracellular matrix
MC: Breast carcinoma
CRC: Colorectal carcinoma
OC: Ovarian carcinoma
PDAC: Pancreatic ductal adenocarcinoma
AdCy: Adenoid cystic carcinoma
MuEp: Mucoepidermoid carcinoma
SaDu: Salivary duct carcinoma
TMA: Tissue microarray
FFPE: Formalin-fixed paraffin-embedded
RNA-ISH: RNA in-situ hybridization
IHC: Immunohistochemistry
MSI: MALDI-TOF–MS-Imaging
TC_*COL11A1*_: Tumour cells with *COL11A1* staining
ER: Oestrogen receptor
Acin: Acinic cell carcinoma
Sec: Secretory carcinoma
EpMy: Epithelial-myoepithelial carcinoma
ANOS: Adenocarcinoma not otherwise specified
Bas: Basal cell adenocarcinoma
MyEp: Myoepithelial carcinoma
FAO: Fatty acid oxidase

## Code Availability

The R code used for statistical analysis is available on request.
