# Correction to: Mutually Exclusive Expression of *COL11A1* by CAFs and Tumour Cells in a Large panCancer and a Salivary Gland Carcinoma Cohort

**DOI:** 10.1007/s12105-021-01379-5

**Published:** 2021-10-06

**Authors:** Christoph Arolt, Franziska Hoffmann, Lisa Nachtsheim, Philipp Wolber, Orlando Guntinas-Lichius, Reinhard Buettner, Ferdinand von Eggeling, Alexander Quaas, Jens Peter Klußmann

**Affiliations:** 1 Medical Faculty, Institute of Pathology, University of Cologne, Kerpener Straße 62, 50937 Cologne, Germany; 2 Department of Otorhinolaryngology, MALDI Imaging and Innovative Biophotonics, Jena University Hospital, 07747 Jena, Germany; 3 Department of Otorhinolaryngology, Head and Neck Surgery, Medical Faculty, University of Cologne, 50937 Cologne, Germany; 4 Department of Otorhinolaryngology, Head and Neck Surgery, Jena University Hospital, 07747 Jena, Germany; 5 MALDI Imaging, Core Unit Proteome Analysis, DFG Core Unit Jena Biophotonic and Imaging, Laboratory (JBIL), Jena University Hospital, 07747 Jena, Germany; 6 Medical Faculty, Centre for Molecular Medicine Cologne (CMMC), University of Cologne, 50937 Cologne, Germany

## Correction to: Head and Neck Pathology 10.1007/s12105-021-01370-0

The original version of this article unfortunately contained a mistake. The peptide sequence (R)ICSINSPGVRPFGAK(D) on pages 3 and 7 is incorrect. The correct sequence is: (R)GEKGEAGPPGAAGPPGAK(G). The authors state that this mistake in displaying the peptide sequence does not affect other results or interpretation of the data. The original article has been corrected.

