# Supplementary material for: Mutually Exclusive Expression of COL11A1 by CAFs and Tumour Cells in a Large panCancer and a Salivary Gland Carcinoma Cohort
Source: Head Neck Pathol. 2021 Aug 10;16(2):394–406. doi: 10.1007/s12105-021-01370-0 (PMC9187800; doi:10.1007/s12105-021-01370-0)
Supplement: Supplementary file 1 — Supplementary file1 (PDF 263 kb) [file 12105_2021_1370_MOESM1_ESM.pdf]

Mutually exclusive expression of COL11A1 by CAFs and tumour cells in a large panCancer and a salivary gland carcinoma cohort

Christoph Arolt 1, Franziska Hoffmann 2, Lisa Nachtsheim 3, Philipp Wolber 3, Orlando Guntinas-Lichius 4, Reinhard Buettner 1, Ferdinand von Eggeling 4,5, Alexander Quaas 1,*, and Jens Peter Klußmann 3,6,*

1 Institute of Pathology, Medical Faculty, University of Cologne, 50937 Cologne, Germany; christoph.arolt@uk-koeln.de (C.A.); reinhard.buettner@uk-koeln.de (R.B.); alexander.quaas@uk-koeln.de (A.Q.)
2 Department of Otorhinolaryngology, MALDI Imaging and Innovative Biophotonics, Jena University Hospital, 07747 Jena, Germany; Franziska.Hoffmann@med.uni-jena.de
3 Department of Otorhinolaryngology, Head and Neck Surgery, Medical Faculty, University of Cologne, 50937 Cologne, Germany; lisa.nachtsheim@uk-koeln.de (L.N.); philipp.wolber@uk-koeln.de (P.W.); jens.klussmann@uk-koeln.de (J.P.K.)
4 Department of Otorhinolaryngology, Head and Neck Surgery, Jena University Hospital, 07747 Jena, Germany; Orlando.Guntinas@med.uni-jena.de (O.G.-L.)
5 MALDI Imaging, Core Unit Proteome Analysis, DFG Core Unit Jena Biophotonic and Imaging, Laboratory (JBIL), Jena University Hospital, 07747 Jena, Germany; Ferdinand.von_Eggeling@med.uni-jena.de (F.v.E.)
6 Centre for Molecular Medicine Cologne (CMMC), University of Cologne, Medical Faculty, University of Cologne, 50937 Cologne, Germany; jens.klussmann@uk-koeln.de (J.P.K.)

Corresponding author: Christoph Arolt, Institute of Pathology, Medical Faculty, University of Cologne; Kerpener Straße 62, 50937 Cologne, Germany; Tel: +49 221 478 4726; Fax: +49 221 478-6360; Email: Christoph.arolt@uk-koeln.de

		CAFs$_{COL11A1}$		
**Organ**	**Histology**	**negative**	**positive**	**n**
Prostate	adeno	96.0%	4.0%	25
	total	96.0%	4.0%	25
Breast	NST	14.3%	85.7%	14
	medullary	0.0%	100.0%	3
	lobular	33.3%	66.7%	3
	tubular	50.0%	50.0%	2
	mucinous	100.0%	0.0%	2
	total	25.0%	75.0%	24
Lung	adeno	78.6%	21.4%	14
	mucinous	100.0%	0.0%	1
	SQC	50.0%	50.0%	6
	LCNEC	66.7%	33.3%	3
	total	70.8%	29.2%	24
Colon	adeno	20.8%	79.2%	24
	total	20.8%	79.2%	24
Cervix	adeno	100.0%	0.0%	6
	SQC	77.8%	22.2%	18
	total	83.3%	16.7%	24
Stomach	diffuse	62.5%	37.5%	8
	mixed	100.0%	0.0%	1
	intestinal	46.7%	53.3%	15
	total	54.2%	45.8%	24
Liver	hepatocellulary	75.0%	25.0%	24
	total	75.0%	25.0%	24
Uterus	serous	50.0%	50.0%	2
	endometrioid	77.3%	22.7%	22
	total	75.0%	25.0%	24
Ovary	serous	64.3%	35.7%	14
	clear	50.0%	50.0%	2
	serous borderline	100.0%	0.0%	3
	mucinous borderline	100.0%	0.0%	4
	total	73.9%	26.1%	23
Esophagus	adeno	63.2%	36.8%	19
	SQC	33.3%	66.7%	6
	total	56.0%	44.0%	25
Lymphnode	DLBCL	80.0%	20.0%	5
	B-CLL	100.0%	0.0%	4
	MZL	100.0%	0.0%	2
	FL	100.0%	0.0%	4
	CHL nodular	100.0%	0.0%	2
	CHL mixed	100.0%	0.0%	2
	plasmocytoma	66.7%	33.3%	3
	T peripheral	100.0%	0.0%	1
	T angioimmun	100.0%	0.0%	1
	T NOS	100.0%	0.0%	1
	total	92.0%	8.0%	25

Supplementary table 1: Percentage of carcinomas from the panCancer cohort with CAFs$_{COL11A1}$ by histologic type. NST: no special type; SQC: squamous cell carcinoma; LCNEC: large cell neuroendocrine carcinoma; DLBCL: diffuse

large B cell lymphoma; B-CLL: B cell chronic lymphatic leukemia; MZL: marginal zone lymphoma; FL: follicular lymphoma; CHL: classic Hodgkin lymphoma; T: T cell

CAFs$_{COL11A1}$ score								
		**0**	**1**	**2**	**3**	**p**	**n**	**spearman coefficient**
Grade	G1	66.7%	33.3%	0.0%	0.0%	**0.021**	24	0.478
	G2	57.1%	0.0%	0.0%	42.9%			
	G3	0.0%	21.4%	42.9%	35.7%			
pT	pT1	37.5%	25.0%	12.5%	25.0%	0.125	24	0.323
	pT2	23.1%	15.4%	30.8%	30.8%			
	pT3	0.0%	0.0%	0.0%	100.0%			
	pT4	0.0%	0.0%	50.0%	50.0%			
Ki 67 (%)	0-9 %	66.7%	33.3%	0.0%	0.0%	**0.001**	22	0.657
	10-19 %	66.7%	0.0%	0.0%	33.3%			
	20-29 %	0.0%	33.3%	0.0%	66.7%			
	30-39 %	0.0%	0.0%	50.0%	50.0%			
	40-49 %	0.0%	0.0%	50.0%	50.0%			
	50-59%	0.0%	0.0%	0.0%	0.0%			
	60-69%	0.0%	0.0%	0.0%	100.0%			
	70-79 %	0.0%	0.0%	50.0%	50.0%			
	80-89 %	0.0%	0.0%	66.7%	33.3%			
	80-99 %	0.0%	0.0%	0.0%	0.0%			
pN	pN0	22.2%	22.2%	22.2%	33.3%	0.554	23	0.133
	pN1	33.3%	0.0%	33.3%	33.3%			
	pN2	0.0%	0.0%	50.0%	50.0%			
	pN3	0.0%	0.0%	0.0%	0.0%			
	total	21.7%(6)	17.4% (4)	26.1% (6)	34.8% (8)			

		**negative**	**positive**	**p**	**n**
histology	NST	14.3%	85.7%	*0.065*	24
	medullary	0.0%	100.0%		
	lobular	33.3%	66.7%		
	tubular	50.0%	50.0%		
	mucinous	100.0%	0.0%		
AR (%)	negative	0.0%	100.0%	0.462	13
	positive	28.6%	71.4%		
ER (%)	negative	0.0%	100.0%	**0.012**	22
	positive	54.5%	45.5%		
PR (%)	negative	14.3%	85.7%	0.137	22
	positive	50.0%	50.0%		
pT	< pT3	28.6%	71.4%	0.546	24
	>= pT3	0.0%	100.0%		
pN	pN-	22.2%	77.8%	1	23
	pN+	20.0%	80.0%		
L	L0	22.2%	77.8%	1	23
	L1	20.0%	80.0%		
V	V0	19.0%	81.0%	NA	21
	V1	0.0%	0.0%		
Pn	Pn0	100.0%	0.0%	NA	21
	Pn1	0.0%	0.0%		
total		25 (6)	75 (18)		

Supplementary table 2: Percentage of breast carcinomas with correlations of $CAFs_{COL11A1}$ and clinicopathological features. Upper half: $CAFs_{COL11A1}$ score in relation to ordinally scaled pathological attributes. Lower half: Comparison of negative (Score 0) and positive (Score >0) cases with respect to nominally scaled clinicopathological parameters. Given as percentage. absolute numbers in brackets. P values below 0.05 are indicated in bold type. p values below 0.1 are marked in italic. P values of the lower half were calculated with fisher's exact test.

CAF$_{SCOL11A1}$ **score**									
		**0**	**1**	**2**	**3**	**4**	**p**	**n**	**spearman coefficient**
Grade	G1	100.0%	0.0%	0.0%	0.0%	0.0%			
	G2	85.7%	0.0%	14.3%	0.0%	0.0%	0.122	15	0.466
	G3	42.9%	14.3%	28.6%	14.3%	0.0%			
pT	pT1	100.0%	0.0%	0.0%	0.0%	0.0%			
	pT2	75.0%	0.0%	25.0%	0.0%	0.0%			
	pT3	75.0%	0.0%	25.0%	0.0%	0.0%	*0.052*	23	0.419
	pT4a	83.3%	16.7%	0.0%	0.0%	0.0%			
	pT4b	25.0%	25.0%	25.0%	25.0%	0.0%			
N	pN0	78.6%	7.1%	14.3%	0.0%	0.0%			
	pN1	75.0%	25.0%	0.0%	0.0%	0.0%	0.390	23	0.185
	pN2	60.0%	0.0%	20.0%	20.0%	0.0%			
CD8	0	87.5%	0.0%	12.5%	0.0%	0.0%			
	1	75.0%	8.3%	8.3%	8.3%	0.0%	0.282	24	0.249
	2	50.0%	25.0%	25.0%	0.0%	0.0%			
total		75 (18)	8.3 (2)	12.5 (3)	4.2 (1)	0 (0)			

		**negative**	**positive**	**p**	**n**
pT	<pT3	80.0%	20.0%	0.653	24
	>=pT3	64.3%	35.7%		
pN	pN-	73.3%	26.7%	1	24
	pN+	66.7%	33.3%		
V	V0	70.6%	29.4%	0.333	18
	V1	0.0%	100.0%		
L	L0	66.7%	33.3%	NA	18
	L1	0.0%	0.0%		
Pn	Pn0	92.3%	7.7%	**0.007**	20
	Pn1	28.6%	71.4%		
Age	< 65y	80.0%	20.0%	0.113	25
	>= 65y	40.0%	60.0%		
Sex	women	68.8%	31.3%	1	25
	men	77.8%	22.2%		
TP53	wildtype	88.9%	11.1%	**0.003**	24
	mutated	16.7%	83.3%		
CD8	not inflamed	76.2%	23.8%	0.548	25
	inflamed	50.0%	50.0%		
total		72.0 (18)	28.0 (7)		

Supplementary table 3: Percentage of adenoid cystic carcinomas with correlations CAFs*COL11A1* and clinicopathological features. Upper half: CAFs*COL11A1* score in relation to ordinally scaled pathological attributes. Lower half: Comparison of negative (Score 0) and positive (Score >0) cases with respect to nominally scaled clinicopathological parameters. Given as percentage. absolute numbers in brackets. P values below 0.05 are indicated in bold type. p values below 0.1 are marked in italic. P values of the lower half were calculated with fisher's exact test.

**CAFs*COL11A1* in cases with nodal metastasis**			**n (%)**
Entity	Acin		1 (7.1)
	ANOS		3 (21.4)
	MuEp		2 (14.3)
	SaDu		8 (57.1)
Status	both positive		5 (35.7)
	both negative		2 (14.3)
	different	Primary positive	5 (35.7)
		Metastasis positive	2 (14.3)

Supplementary table 4: Concordance of CAFs*COL11A1* positivity between primaries and lymphnode metastases for salivary gland carcinomas. acin: acinic cell carcinoma; ANOS: adenocarcinoma not otherwise specified; MuEp: mucoepidermoid carcinoma; SaDu: salivary duct carcinoma

	**TC*COL11A1***	**n**
Myoepithelial	42.2	4
Epithelial-Myoepithelial	29.6	6
Basal cell	16.9	4
Adeno NOS	14.6	6
Adenoidcystic	13.5	25
Acinic cell	10.6	10
Mucoepidermoid	1.2	29
Secretory	0.1	7
Salivary duct	0.0	19
Mean overall	8.9	

Supplementary table 5: Mean percentage of TC*COL11A1* per SGC type.
